# Financial risk of seeking maternal and neonatal healthcare in southern Ethiopia: a cohort study of rural households

**DOI:** 10.1186/s12939-020-01183-7

**Published:** 2020-05-18

**Authors:** Moges Tadesse Borde, Eskindir Loha, Kjell Arne Johansson, Bernt Lindtjørn

**Affiliations:** 1 School of Public Health, College of Medicine and Health Sciences, Hawassa University, P.O. Box 1436, Hawassa, Ethiopia; 2 Centre for International Health, University of Bergen, Bergen, Norway; 3 School of Public Health, College of Medicine and Health Sciences, Dilla University, Dilla, Ethiopia; 4 Department of Infectious Disease Epidemiology, London School of Hygiene and Tropical Medicine, London, UK; 5 Department of Global Public Health and Primary Care, University of Bergen, Bergen, Norway

**Keywords:** Financial risk, Maternal and neonatal healthcare, Southern Ethiopia, Cohort study, Rural households

## Abstract

**Introduction:**

Ethiopian households’ out-of-pocket healthcare payments constitute one-third of the national healthcare budget and are higher than the global and low-income countries average, and even the global target. Such out-of-pocket payments pose severe financial risks, can be catastrophic, impoverishing, and one of the causal barriers for low utilisation of healthcare services in Ethiopia. This study aimed to assess the financial risk of seeking maternal and neonatal healthcare in southern Ethiopia.

**Methods:**

A population-based cohort study was conducted among 794 pregnant women, 784 postpartum women, and their 772 neonates from 794 households in rural *kebeles* of the *Wonago* district, southern Ethiopia. The financial risk was estimated using the incidence of catastrophic healthcare expenditure, impoverishment, and depth of poverty. Annual catastrophic healthcare expenditure was determined if out-of-pocket payments exceeding 10% of total household or 40% of non-food expenditure. Impoverishment was analysed based on total household expenditure and the international poverty line of ≈ $1.9 per capita per day.

**Results:**

Approximately 93% (735) of pregnant women, 31% (244) of postpartum women, and 48% (369) of their neonates experienced illness. However, only 56 households utilised healthcare services. The median total household expenditure was $527 per year (IQR = 390: 370,760). The median out-of-pocket healthcare payment was $46 per year (IQR = 46: 46, 92) with two episodes per household, and shared 19% of the household’s budget. The poorer households paid more than did the richer for healthcare, during pregnancy-related and neonatal illness. However, the richer paid more than did the poorer during postpartum illness. Forty-six percent of households faced catastrophic healthcare expenditure at the threshold of 10% of total household expenditure, or 74% at a 40% non-food expenditure, and associated with neonatal illness (aRR: 2.56, 95%CI: 1.02, 6.44). Moreover, 92% of households were pushed further into extreme poverty and the poverty gap among households was 45 Ethiopian Birr per day. The average household size among study households was 4.7 persons per household.

**Conclusions:**

This study demonstrated that health inequity in the household’s budget share of total OOP healthcare payments in southern Ethiopia was high. Besides, utilisation of maternal and neonatal healthcare services is very low and seeking such healthcare poses a substantial financial risk during illness among rural households. Therefore, the issue of health inequity should be considered when setting priorities to address the lack of fairness in maternal and neonatal health.

## Background

Among the primary objectives of healthcare systems are to treat sick people and protect them from financial risk [1]. Tax systems and health insurance are major mechanisms that pool financial risk and assure more predictable healthcare finances [2]. However, households with tight financial constraints in low and middle-income countries (LMICs) still pay high levels of direct out-of-pocket (OOP) healthcare payments during illness [3] at the point of seeking healthcare [4]. Moreover, there is low coverage or utilisation of healthcare services during illness, and OOP healthcare payments could be one of the causal barriers. The high OOP healthcare payments prevent patients from seeking essential healthcare. Furthermore, there is limited evidence on the level of financial risk due to OOP healthcare payments for illness during pregnancy, postpartum, and neonatal periods in rural Ethiopia and such evidence is needed for creating fair health policies [5].

Financial risks are financial catastrophes and impoverishment due to OOP healthcare payments [6]. A household’s capacity to pay is the net remaining after expenditure on essential goods (i.e., non-food expenditure) and used as a proxy measure for a household’s ability to pay [7]. Globally, each year, it is estimated that more than 150 million individuals from 44 million households face catastrophic healthcare expenditures (CHEs) and that more than 100 million individuals from 25 million households are pushed into extreme poverty due to OOP healthcare payments [8]. Financial risks might force households to cut their basic necessities, and sell assets [6]. Moreover, poor households may not even be able to afford to seek essential healthcare and they remain trapped in a vicious circle of illness and poverty [6, 9].

Previous studies indicated that OOP healthcare payments were high during illness. For example, the OOP healthcare payment for sick postpartum women in Bangladesh was $261 [10]. OOP healthcare payments accounted for 40% in Chile [11], more than 50% for Indian sick neonates [12], and they were three times higher during hospitalisation among the poorest Indian households [13]. Approximately 4 to 6% of households in Vietnam faced CHE [14], 2 to 3% in Iran [15], and 2 to 28% in Kenya [5]. Due to high CHE, people would likely forgo the healthcare that they need, as they could not afford it. Possible influencing factors were household economic status, educational status, and occupation of the head of the household [16]. Increasing domestic investments in public healthcare finance can reduce the risks involved in OOP healthcare payments [17]. Besides, incorporating financial risk protection mechanisms into the healthcare system [18], and reforms towards universal health coverage [19], could substantially improve the health status of households.

In Ethiopia, 31–34% of the national healthcare budget (total health expenditure) was financed by OOP healthcare payments (2010/11–2016/17) [9, 20, 21], which is considerably higher than 21% of the global average, 15–20% of the global target, and even higher than 30% of the low-income countries average [21, 22]. Such high OOP healthcare payments for healthcare result in severe financial risks and can be catastrophic and impoverishing for poor households. In 2013, in Ethiopia, it was estimated that 350,000 poverty cases were due to direct OOP medical costs [23]. Approximately 7% of Ethiopian households with children suffered from severe pneumonia. Furthermore, approximately 6% of Ethiopian households with severe diarrhoea were pushed into extreme poverty and poorer and rural households were more likely to be impoverished due to OOP healthcare payments for these services [24]. These findings indicate that OOP healthcare payments are highly linked to financial risks [20].

The Ethiopian government is attempting to remove financial barriers associated with seeking healthcare, reduce catastrophic OOP healthcare payments, and increase utilisation of healthcare services by scaling-up health insurance schemes in the following major ways: community-based health insurance (CBHI) for informal sectors of the economy in urban and rural areas, which now covers over 22.5 million citizens [25]; and social health insurance (SHI) for civil servants and the formal sector, which is currently about to be launched by the government [26]. However, poor mothers and neonates, with a high rate of illness, are still making a considerable amount of OOP healthcare payments [9] because community-based health insurance schemes are not yet in place in the study area.

Based on these findings, we hypothesized that households faced high CHE and poverty due to healthcare seeking. Secondly, we aimed to elucidate to what extent OOP healthcare payments influenced healthcare utilisation and related coping mechanisms. Therefore, we attempted to fill this knowledge gap and to assess the financial risk of seeking maternal and neonatal healthcare during an illness of pregnancy, postpartum, and neonatal periods in southern Ethiopia.

## Methods and materials

### Study setting and population

In this study, a population-based cohort study was conducted among 794 pregnant women, 784 postpartum women, and their 772 neonates from 794 rural households to estimate CHE due to illness during pregnancy, postpartum, and neonatal periods. A household was designated as consisting of individuals who lived in the same dwelling and who had common arrangements for basic domestic and/or reproductive activities.

This study was performed in three randomly selected *kebeles* (i.e., *Mekonisa, Hase-Haro, and Tumata-Chiricha*) from the *Wonago* district of southern Ethiopia, which is located 420 km from the capital city of Addis Ababa. The data were collected from May 2017 to July 2018 for 15 months. The study area comprised four health posts and two health centers with a total population of almost 29,000 [27]. In 2013, more than 80% of the Ethiopian population lived in a rural area, 26% of residents earned less than $1 per day, and 77% of rural women travelled more than 20 km to reach a hospital [28]. Detailed information on the methods, study design, procedure, sample size, and major findings were presented in our previous study [29].

### Sample size and sampling technique

**Fig. 1:**
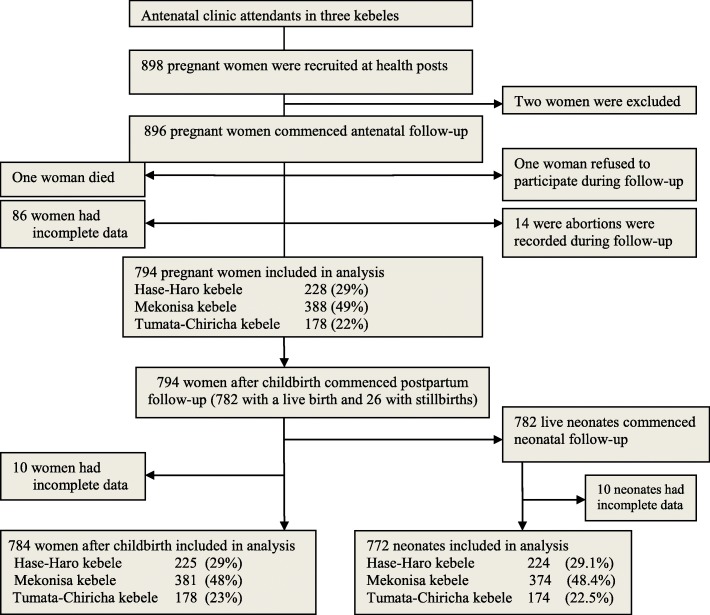
Flowchart of recruitment of pregnant women, postpartum women, and their neonates in rural southern Ethiopia, May 2017 to July 2018

The sample size was determined by Openepi software Version 3.03 (www.openepi.com) for epidemiological studies [29]. This sample size was also used for the economic evaluation. We assumed 15.5% of the incidence of pregnancy-related illness, and a 1.65 relative risk [30] among poor women, compared with rich women (95% confidence level, 80% power, and 1:1 ratio of unexposed to exposed). After adding 10% of non-response, the sample size was estimated to be 898 (Fig. 1). Each participant was visited at home (i.e. every two weeks for pregnant women, eight times for postpartum women up to 42 postpartum days, and six times for their neonates up to the age of 28 days). First, pregnant women were recruited from health posts attending antenatal care and interviewed about socioeconomic and demographic characteristics. Included participants were those who participated in respective pregnancy-related, postpartum, and neonatal illness study, and identified during scheduled visits. Those study participants who were not easily contacted or those who presented with illness after arranged pregnancy, postpartum and neonatal visit days were excluded.

### Outcome variables

The primary outcome variables were the catastrophic and/or impoverishing effect of OOP healthcare payments. CHE was dichotomized with a value of 0 or 1 (0 = not facing CHE, and 1 = facing CHE). Impoverishment was also dichotomized with a value of 0 or 1 (0 = not impoverished, 1 = impoverished) [31].

### Exposure variables

The exposure variables concerned the socioeconomic status of the household. The variables included: (1) predisposing factors: utilisation of healthcare services during illness, household size, and age of pregnant women; (2) enabling factors: socio-demographic characteristics (educational status and occupation of the head of the household, and households’ wealth quintiles); and (3) need factors: illness among pregnant women, postpartum women, and neonates. In this study, we used total household expenditure, instead of household income, as consumption is a better proxy for household welfare level in low-income settings [32].

### Data collection tools and quality assurance

Baseline socio-economic and follow-up data were collected via an interviewer-administered questionnaire during visits to the participants’ homes. The questionnaire was adapted from an earlier survey in Ethiopia [33], and the data collection was guided by published techniques and their implementation to analyse health equity using household survey data [31]. The questionnaire was prepared in English, translated into the local *Gedeo* (see Additional file 1.txt) and Amharic languages (see Additional file 2.txt), and then translated back into English (see Additional file 3.txt). A pre-test was conducted in a neighboring *kebele.* Data collectors read the questions aloud and asked the women to indicate whether they had any symptoms of pregnancy-related, postpartum, and neonatal illness; whether they utilised healthcare services; the amount of OOP healthcare payments made by the household, and sources of coping mechanisms concerning OOP healthcare payments. The data collectors were trained women, residents of the selected *kebeles*, and had completed at least grade 10. The data collectors and supervisors were experienced in data collection and supervision.

### Patient payment for healthcare

Patient payment for utilisation of healthcare services during maternal and neonatal illness was calculated by summation of the household’s *direct medical* and *direct non-medical* OOP healthcare payments. A household’s *direct medical* OOP healthcare payment was calculated in terms of direct payment made by households to healthcare providers at the point of receiving healthcare services due to illness. This included registration/card fees, medicines, laboratory tests, etc., for outpatient visits; and for inpatient stays, bed charges at healthcare facilities. *Direct medical* OOP healthcare payment also excluded any prepayment for healthcare services, i.e. taxes or insurance. Household’s *direct non-medical* OOP healthcare payment was calculated in terms of payments related to transportation, and daily living payments including accommodation, and food for the accompanying household members or caregivers, and additional expenses for the caregiver during outpatient and inpatient visits [7]. The reference period for outpatient and inpatient payments was one year (12 months). Even though we did not collect on informal (“envelope”) payments for healthcare services, there are several problems in Ethiopia, including informal healthcare provision, illicit charging, and corruption [34].

All estimates for annual total household expenditure and OOP healthcare payments were self-reported. Besides being convenient, self-report of these estimates have demonstrated to be effective in capturing household expenditure and OOP healthcare payments. However, there could be over or under-reporting. To avoid over or under-reporting, we used short recall visit time [31].

Both total household and non-food expenditures were used to measure the incidence and intensity of catastrophic payments and their impacts on poverty. Total household expenditure was used to construct the quintiles for households as a direct measure of the living standard of the households. Poverty differences were shown across the quintiles of total household expenditures and between gross and net of healthcare payments. On this basis, the households were classified into five quintiles and were designated from the lowest to the highest quintiles. Financial fairness (equity) was estimated by measuring the relationship between OOP healthcare payments and the ability to pay. A percentage of OOP healthcare payments with total household expenditure by quintile of total household expenditure was estimated to assess the distribution of economic benefits and burdens in society [31].

Total household expenditure was calculated by summation of all expenditures on food and non-food expenditures. It comprised the monetary value of the consumption of home-made products [7] and computed based on 10 different types of household expenditures. For food and supplies, the head of the household was asked, “On average, about how much have you spent in ETB per day?”; for all other expenditure categories, the survey question was phrased, “About how much did you spend?”; for utilities, in ETB per month (i.e., for electricity, water, and telephone service); for goods and utensils, in ETB per year; for education, in ETB per semester (i.e., for children or self); for OOP healthcare payments, in ETB in the last three months as baseline data; for house rent, in ETB per month; for clothes, in ETB per year; for maintenance of bicycles, carts, motorbikes, etc., in ETB per month; for replacements of household appliances, in ETB per month; and for reimbursement of the loan(s), in ETB per month [35]. All expenditures were collected in local currency or Ethiopian ETB and then converted to United States dollars ($). The average 2017/18 exchange rate of $1 was equal to 26.11 ETB [36]. In this study, financial risk due to OOP payments for seeking healthcare for maternal and neonatal illness was estimated using the following four indicators of financial risk protection (FRP): incidence of CHE, mean positive catastrophic overshoot, the incidence of impoverishment, and increment of the depth of poverty [31].

To estimate the proportion of households incurring CHE, OOP healthcare payment(s) by each household was divided by total household expenditure per year and reported as a percentage. CHE was defined as OOP healthcare payments that became catastrophic if the OOP healthcare payment exceeded a 10% threshold of total household expenditure or a 40% threshold of non-food expenditure (capacity to pay) [37]. The fraction of households’ OOP healthcare payment to total household expenditure (at 10%), or capacity to pay (at 40%) × 100 [38] was used for estimation of the variability of the financial burden. To derive households’ total annual OOP healthcare payments, we normalized expenditures to an annual scale in 12 months, and then summed across categories.

To assess the impoverishing effects of OOP healthcare payments, the incidence of CHE was estimated using poverty headcount. Poverty headcount was estimated by the proportion or ratio of households that incurred catastrophic OOP healthcare payments that exceeded the defined threshold. The intensity of CHE was also assessed using overshoot and using mean positive overshoot [37]. Overshoot was measured using the average percentage of households which incurred catastrophic OOP healthcare payments and exceeded the threshold across the entire sample. Mean positive overshoot was the average percentage of households which incurred catastrophic OOP healthcare payments and that exceeded the threshold, but only among households that exceed either threshold. The poverty impact was estimated using poverty headcount including gross of and excluding net of OOP healthcare payments [39] and the poverty gap using the poverty line [40].

In this study, Pen’s Parade plot was produced to illustrate the magnitude of impoverishment, using plots of two expenditure parades (i.e., total household expenditure and such expenditure net of OOP healthcare payments), with a cumulative proportion of households ranked according to their total household expenditure [31]. Therefore, impoverishment was analysed based on total household expenditure gross of and net of OOP healthcare payments and the international poverty line, PPP $1.9 ≈ 49.6 ETB per capita per day, using the 2015/16 report on poverty and household welfare from Ethiopia [41]. The coping mechanism employed for financial difficulties by households to cover OOP healthcare payment(s) was also analysed (i.e., selling of assets, and borrowing).

### Statistical analysis

The data were entered in EpiData version 3.1 software (EpiData Association Odense, Denmark). For analysis of financial risks (i.e., catastrophe and impoverishment), three variables were used: OOP healthcare payments, total household expenditure, and non-food expenditure. Our study used the households as the unit of analysis. The OOP healthcare payment was disaggregated by pregnancy-related, postpartum, and neonatal illness. The concentration curve was used to measure inequality in the distribution of total household expenditures, OOP healthcare payments, and utilisation of healthcare services. The concentration curve laid inside and/or outside the per capita total household expenditure curve (Lorenz curve) gross OOP healthcare payments. The farther is the curves from the 45° line of equality, the greater is the inequality [31].

Univariate analysis was conducted using descriptive analysis. Then, bivariate analysis was carried out to analyse the difference between variables. As per the recommendation of Hosmer and Lemeshow, variables with P -values ≤0.2 in univariate analysis were used for multivariate analysis [42]. *P*-values ≤0.05 were used as cut-off points to determine significant association. Multivariate logistic regression analysis was performed to identify factors associated with CHE. The strength of these associations was quantified using odds ratio (aRR) with corresponding 95% confidence intervals (CI). Data were analysed using SPSS software, version 25 (SPSS Inc. Chicago, IL, U.S.A.), and Automated Development Economics and Poverty Tables (ADePT) software, version 6.06648 developed by World Bank’s experts (www.worldbank.org/adept).

## Result

A total of 896 households of pregnant women were recruited. Of these, 11% (102 of 896 women) had incomplete data and were excluded (i.e., 86 women dropped-out, one died, one refused to participate, and 14 abortions occurred after week 21 and before 28 weeks of gestation). In the analysis, 794 pregnant women, 784 postpartum women, and their 772 neonates were included from 794 households (Fig. 1). The response rate was 89% (794 of 896 women). The average household size among study households was 4.7 persons per household.

### Household characteristics

Table 1 presents the socio-economic characteristics and illness status of the 794 households. From enabling factors, 167 (21%) of heads of households had no formal education. Of the need factors, 93% of pregnant women (735 of 794), 31% of postpartum women (244 of 784), and 48% of neonates (369 of 772) experienced an illness during the study period. However, only 56 households utilised healthcare services (i.e., 6%, 41 of 735 of sick pregnant women; 2%, 5 of 244 of sick postpartum women; and 3%, 10 of 369 of sick neonates). Concerning predisposing factors, 68% (537 of 794) of the total household expenditure was below the poverty line of $1.9 per day or $693.5 per year, and 71% (560 of 794) of food expenditure was also below the poverty line of $1.9 per day.

**Table 1:** Characteristics of households in rural southern Ethiopia, May 2017 to July 2018

Household’s characteristics		Frequency	Percent
Kebele/residence (*n* = 794)	Mekonisa	388	49
	Hase-Haro	228	29
	Tumata-Chiricha	178	22
Age of pregnant women (n = 794)	15–19	106	13
	20–24	226	29
	25–29	289	36
	30–34	131	17
	35+	42	5
**Predisposing factors**			
Pre-payment total household expenditure per year in $ (n = 794)	< $693.5	537	68
	$693.5+	257	32
Post-payment total household expenditure per year in $ (n = 794)	< $693.5	552	70
	$693.5+	242	30
Food expenditure per year in $ (n = 794)	< $693.5	560	71
	$693.5+	234	29
Non-food expenditure per year in $ (n = 794)	< $693.5	787	99
	$693.5+	7	1
**Utilisation of healthcare services during illness**			
Pregnant women (*n* = 735)	Yes	41	6
	No	694	94
Postpartum women (*n* = 244)	Yes	5	2
	No	239	98
Neonates (*n* = 369)	Yes	10	3
	No	359	97
**Enabling factors**			
Educational status of the head of the household (n = 794)	No education	167	21
	Primary	470	59
	Secondary and above	157	20
Occupation of the head of the household (n = 794)	Agriculture	471	59
	Sales and services	45	6
	Skilled manual	22	3
	Professional/technical/managerial	22	3
	Unskilled manual	159	20
	Others	75	9
**Need factor:** illness occurrence			
Pregnant women (n = 794)	Yes	735	93
	No	59	7
Postpartum women (*n* = 784)	Yes	244	31
	No	540	69
Neonates (*n* = 772)	Yes	369	48
	No	403	52

**Note:** 1 Ethiopian ETB equals 0.0383 dollars ($1 = ETB 26.11)

### Household expenditures

Table 2 presents household expenditures per year. There were more observations below and above the mean (right-skewed) for both total and non-food expenditures. The median total households’ expenditure of $527 (13,760 ETB) per year (IQR = 390: 370,760). The median households’ non-food expenditure was $67 (1749 ETB) per year (IQR = 46: 46, 92), and accounted for 15.7% of households’ budget (95%CI: 15.6, 15.8). Households’ budget share on food was 84.3% (95%CI: 84.2, 84.4).

**Table 2:** Household expenditures per year in southern Ethiopia, 2017/2018 (n = 794)

Expenditures	Mean per household ($)	Median per household ($)	Total ($)
Food and supplies **(**i.e., food, plates, cups, etc. which were bought, grown or produced, received as wages, received as a gift or loan, or spent on cooking and lighting fuel)	500	419	401,992
Utilities (i.e., electricity, water, telephone, etc)	12	14	9861
Education (i.e., schooling for children or self)	2	0	1383
House rent	7	0	5127
Goods and utensils for household use	8	8	6596
Clothes	32	27	25,578
Maintenance of bicycle(s), carts, motorbike, etc	10	0	8065
Replacements of household appliances (i.e., stove, lanterns, etc.)	13	14	10,227
Reimbursement of loan (s)	10	0	7848
Pre-healthcare total household expenditure (gross)	593	527	476,677
Post- healthcare total household expenditure (net)	576	503	462,875
Non-food expenditure (i.e., ability to pay)	93	67	74,685
The proportion of expenditure on food to total household expenditure	84%	88%	84%

### Out-of-pocket (OOP) healthcare payments

There were 109 episodes of out-of-pocket healthcare payment with two episodes per household (i.e., 109 episodes per 56 households). However, the episode of OOP healthcare payments during pregnancy-related illness was 1.2 episodes per household (i.e., 51 episodes per 41 sick pregnant women), 8.2 episodes during postpartum illness (i.e., 41 episodes per five sick women after childbirth), and 1.7 episodes during neonatal illness (i.e., 17 episodes per 10 sick neonates).

The total OOP healthcare payment during illness was $13,802 (360,370 ETB) per year with median of $46 (1202 ETB) per household (IQR = 46: 46, 92) (Table 3). However, on average, OOP healthcare payment for direct medical services was $105.5 and $21.5 for non-medical expenses. The average OOP healthcare payment during pregnancy-related illness (*n* = 51) was $95.6 (i.e., $89.1 for direct medical and $6.5 for direct non-medical expenses). The average OOP healthcare payment during postpartum illness (*n* = 41) was also $22.7 (i.e., $15.4 for direct medical and $7.3 for direct non-medical expenses). Besides, the average OOP healthcare payment during neonatal illness (*n* = 17) was $8.9 (i.e., $1.1for direct medical and $7.8 for direct non-medical expenses).

#### Per capita healthcare finance across quintiles

Table 3: presents per capita healthcare finance across quintiles of total household expenditure. The median per capita total household expenditure for the lowest, second and third quintile was lower than the total median, $527 (13,760 ETB), which indicated that more than 40% (343 of 794) of households consumed less than the median. Per capita total household expenditure gross of OOP healthcare payments for the lowest quintile was $73; while the net of OOP healthcare payment was $69. The median per capita total household expenditure in the lowest quintile ($73) was less than half of the total median ($527). Total median per capita total household expenditure among the lowest quintile was 14% ($73 of $527); however, it was 40% in the highest quintile ($212 of $527).

The lowest quintile contributed to $7 OOP healthcare payments, which was less than half that of the highest quintile ($15). Households in the lowest quintile consumed 0.34 times that of per capita total household expenditure to the highest quintile in respect of gross of ($73/$212) and net of ($69/$203) of OOP healthcare payments, which indicated that inequity existed in OOP healthcare payments between the lowest and the highest quintiles.

**Table 3:** Per capita healthcare finance across quintiles of total household expenditure in southern Ethiopia, 2017/2018

Quintiles	Per capita annual total household expenditure, (gross of)		Household annual OOP healthcare payments		Per capita annual total household expenditure, (net of)	
	Mean ($)	Median ($)	Mean ($)	Median ($)	Mean ($)	Median ($)
Lowest quintile	82	73	20	7	80	69
Second quintile	83	73	22	8	80	70
Third quintile	89	79	21	8	86	75
Fourth quintile	101	90	22	8	98	86
Highest quintile	239	212	42	15	232	203
Total	593	527	127	46	576	503

**Note:** 1 Ethiopian ETB equals 0.0383 dollars ($1 = 26.11ETB)

#### Out-of-pocket (OOP) healthcare payment share across quintiles

**Fig. 2:**
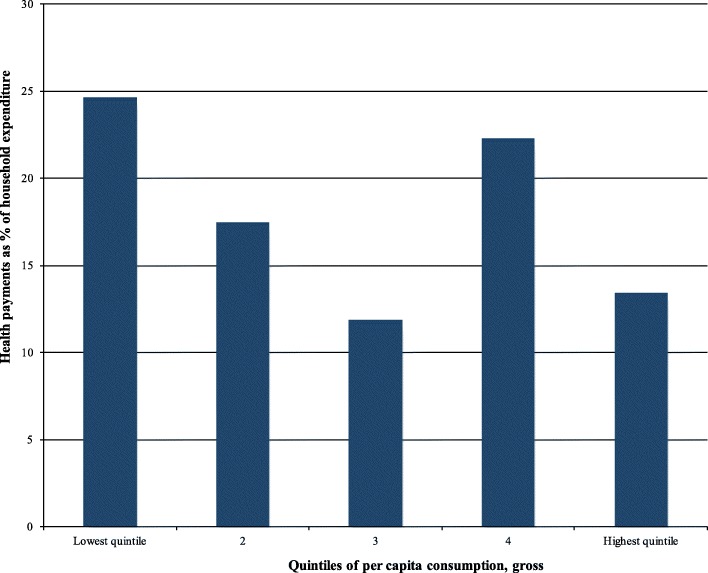
Healthcare payment shares by quintiles in rural southern Ethiopia, 2017/18

Figure 2 presents the OOP healthcare payments share by quintiles. The financing budget share of OOP healthcare payments to quintiles of per capita total household expenditure or consumption decreased from the lowest quintile to the third quintile. In the lowest quintile, the household’s budget share of OOP healthcare payment was 22.4%. On the other hand, it was 18% in the second; 11.5% in the third; 22.2% in the fourth; and 19% in the highest quintile. In general, the overall household’s budget share of total OOP healthcare payments across quintiles was 18.6%.

### Catastrophic healthcare expenditures (**CHE)**

Table 4 presents the incidence (headcount) and intensity of CHE (overshoot and mean positive overshoot) at thresholds of total household (non-food) expenditures. At a 10% threshold of total household expenditure, the incidence of CHE was 45.6% (50 of 109 households) (i.e., the proportion of households whose budget share for OOP healthcare payment exceeded the threshold), the overshoot was 10.4%, (i.e., average excess OOP healthcare payment budget share among all of the households), and the mean positive overshoot was 22.8% (i.e., average excess OOP healthcare payment budget share of those households with CHE). However, at a 40% non-food expenditure threshold, the incidence of CHE was 74.4% (81 of 109 households), the overshoot was 202.7%, and the mean positive overshoot was 272.5%.

**Table 4:** Incidence and intensity of CHE in southern Ethiopia, 2017/2018

Total household expenditure	Threshold budget share			
	10%	15%	25%	40%
CHE headcount	45.6	29.0	17.1	11.5
**Overshoot**	10.4	8.7	6.5	4.4
**Mean positive overshoot**	22.8	29.9	38.2	38.2
**Non-food expenditure**				
CHE headcount	–	–	84.4	74.4
**Overshoot**	–	–	214.7	202.7
**Mean positive overshoot**	–	–	254.4	272.5

Table 5 presents the incidence of CHE among poor and rich households. The negative concentration index for CHE showed a greater tendency for the poor to cross the CHE threshold.

**Table 5:** Incidence of CHE among poor and rich households in southern Ethiopia, 2017/2018

Concentration indexes for:	Threshold budget share			
	10%	15%	25%	40%
CHE (relative to total household expenditure)	−0.114	−0.099	−0.078	−0.052
CHE (relative to non-food expenditure)	–	–	−0.264	−0.275

### Coping strategies

Coping strategies adopted based on the extent of a financial burden on households and thus depend on the burden of maternal and neonatal illness and the rate utilisation of healthcare services. About 13% of households (7 of 56) employed different strategies to cope with financial hardship to cover OOP healthcare payments; including loan or borrowing from family members, 4% (2 of 56 households); loan or borrowing from neighbours, 4% (2 of 56 households); and loan or borrowing from friends with interest, 6% (3 of 56 households).

### Impoverishing catastrophic healthcare expenditures

Table 6 presents the analysis of impoverishment based on expenditure gross of and net of OOP healthcare payments. Approximately 99.6% of households were living below the poverty line after healthcare expenditure. The average deficit or depth of poverty to reach the poverty line was 45.4 Ethiopian Birr per day. Moreover, 91.6% of households were pushed further below the poverty line due to CHE. The increase in poverty due to CHE or the percentage of point change according to the poverty headcount was 0.3 (0.3%) and 0.9 (2%) according to the poverty gap.

**Table 6:** Analysis of impoverishment based on total household expenditure gross of and net of out-of-pocket healthcare payment (poverty line = PPP $1.9 ≈ 49.6 ETB) in southern Ethiopia, 2017/2018

Analysis of impoverishment	Gross of OOP healthcare payment	Net of OOP healthcare payment
Poverty headcount (%)	99.3	99.6
Poverty gap (ETB)	44.5	45.4
Normalized poverty gap (% of the poverty line)	89.7	91.6
Normalized mean positive poverty gap (% of the poverty line)	90.4	92.0

**Fig. 3:**
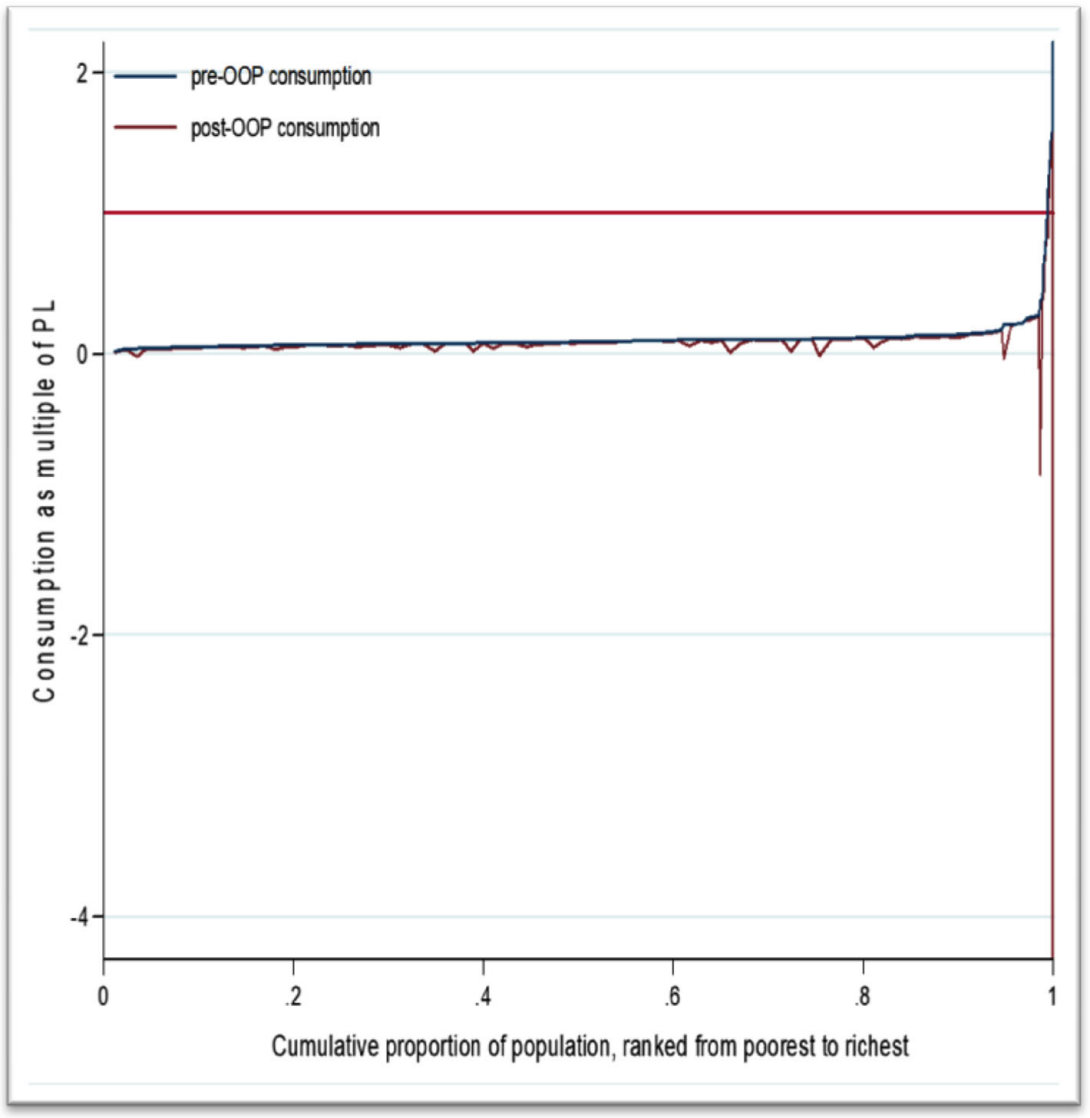
Pen’s Parade of total household expenditure gross of and net of out-of-pocket healthcare payments during pregnancy-related, postpartum, and neonatal illness in rural southern Ethiopia, 2017/18

The Pen’s Parade quintile diagram illustrated the magnitude of impoverishment due to CHE. On the horizontal axis, every household was arranged from the poorest to richest, while the vertical axis showed the level of OOP healthcare payments per capita. The bold red flat line in the figure was the international poverty line. The two important findings from the plot were that there were extremely poor households living below the poverty line, and there was poor utilisation of healthcare, as the incidence of OOP healthcare payments was low which was indicated in the few red drops. Even if the welfare of households was increasing among currently rich households, the extent and depth of poverty were also increased (Fig. 3).

### Concentration curve

**Fig. 4:**
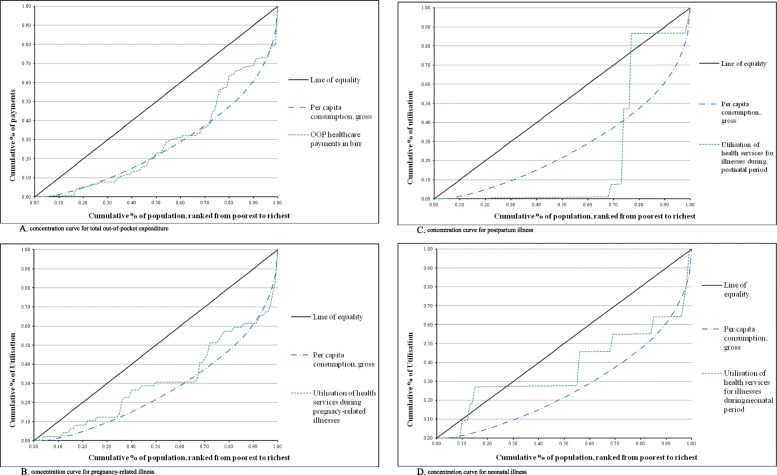
A. concentration curve for out-of-pocket healthcare payments in ETB; 4.B. concentration curve for utilisation of healthcare services during pregnancy-related illness; 4.C. concentration curve for utilisation of healthcare services during the postpartum illness; and 4.D. concentration curve for utilisation of healthcare services during the neonatal illness in rural southern Ethiopia, 2017/18

Fig. 4 A., B., C., and D. present the concentration curves for OOP healthcare payments and utilisation of available healthcare services.

Figure-4. A. shows the concentration curve for OOP healthcare payments. Comprising up to 40% of consumption, the concentration curve of OOP healthcare payments lay outside per capita gross consumption (Lorenz curve). This indicated that the rich households paid more of their total household expenditure for healthcare than did the poor households. However, after 40% of consumption, the concentration curve of OOP healthcare payments was located inside the per capita gross consumption curve. This suggested that the poorer paid more than did the richer for healthcare.

Figure-4. B. presents the concentration curve for the utilisation of healthcare services during pregnancy-related illness almost all lay inside the per capita gross consumption curve. This indicated that the poorer households paid more than did the richer for utilisation of healthcare services during pregnancy-related illness.

Figure-4. C. shows the concentration curve for the utilisation of healthcare services during the postpartum illness with an abnormal peak just after the 65% mark of the population ranked from the poorer to richer, and was located almost outside the per capita gross consumption curve. This suggested that the richer households paid more than did the poorer for utilisation of healthcare services during postpartum illness.

Figure-4. D. presents the concentration curve for the utilisation of healthcare services during neonatal illness with an abnormal peak just after the 20% mark of the population ranked from poorest to richest, and almost all of the concentration curve of the utilisation of healthcare services during neonatal illness was located inside the per capita gross consumption curve. This suggested that the poorer households paid more than did the richer for utilisation of healthcare services during neonatal illness.

### Determinants of catastrophic healthcare expenditures

Table 7 shows the determinants of CHE. Households with neonatal illness were three times more likely to experience CHE than those households without neonatal illness (aRR: 2.56, 95%CI: 1.02, 6.44).

**Table 7:** Determinants of catastrophic healthcare expenditure in rural southern Ethiopia, May 2017 to July 2018

Household’s characteristics		Catastrophic headcount											
		At a 10% threshold of total household expenditure						At a 40% threshold of non-food expenditure					
		Yes	No	Crude relative risk (95.0%CI)	p-value	Adjusted relative risk (95.0%CI)	p-value	Yes	No	Crude relative risk (95.0%CI)	p-value	Adjusted relative risk (95.0%CI)p-	p-value
**Utilisation of healthcare services during:**													
Pregnancy-related illness (41 of 735)	No	46	53	1.30 (0.35,4.90)	0.696	–	–	74	25	1.27 (0.31,5.28)	0.744	–	–
	Yes	4	6	1.0		–	–	7	3	1.0		–	–
Postpartum illness (5 of 244)	No	45	9	–	–	–	–	76	28	–	–	–	
	Yes	4	0	–	–	–	–	4	0	–	–	–	–
Neonatal illness (10 of 369)	No	43	58	–	–	–	–	73	28	–	–	–	–
	Yes	2	0	–	–	–	–	2	0	–	–	–	–
**Enabling factors**													
Educational status of the head of the household	No education	8	7	2.29 (0.63,8.32)	0.210	–	–	11	4	1.38 (0.34,5.56)	0.655	–	–
	Primary	33	34	1.94 (0.76,4.93)	0.163	–	–	52	15	1.73 (0.65,4.64)	0.274	–	–
	Secondary and above	9	18	1.0		–	–	18	9	1.0		–	–
Occupation of the head of the household	Agriculture	30	38	0.34 (0.08,1.42)	0.139	–	–	57	11	–	–	–	–
	Sales and services	4	2	0.86 (0.10,7.51)	0.889	–	–	5	1	–	–	–	–
	Skilled manual	1	2	0.21 (0.01,3.37)	0.273	–	–	3	0	–	–	–	–
	Professional/technical/managerial	1	4	0.11 (0.01,1.41)	0.089	–	–	2	3	–	–	–	–
	Unskilled manual	7	10	0.30 (0.06,1.58)	0.156	–	–	7	10	–	–	–	–
	Others	7	3	1.0		–	–	7	3	–	–	–	–
**Illness among:**													
Pregnant women (735 of 794)	No	5	3	2.07 (0.47,9.15)	0.335	–	–	8	0	–	–	–	–
	Yes	45	56	1.0		–	–	73	28	–	–	–	–
Postpartum women (244 of 784)	No	31	36	1.10 (0.50,2.40)	0.811	–	–	50	17	1.08 (0.45,2.61)	0.867	–	–
	Yes	18	23	1.0		–	–	30	11	1.0		–	–
Neonates (369 of 772)	No	35	39	1.71 (0.70,4.16)	0.241	–	–	58	16	2.56 (1.02,6.44)	0.046	2.56 (1.02,6.44)*	0.046
	Yes	10	19	1.0		–	–	17	12	1.0		1.0	

^*^Adjusted for postpartum and neonatal illness, and utilisation of healthcare services for pregnancy-related illness, and educational status of head of the household

## Discussion

This study indicated that a significant proportion of the households experienced CHE and were forced below the poverty line due to OOP healthcare payments for pregnancy-related, postpartum, and neonatal illness among rural households in southern Ethiopia. This is evidenced that unless the OOP healthcare payments fall to 15–20% of total health expenditures, the incidence of financial catastrophe and impoverishment could not fall to negligible levels [22].

Moreover, households were very poor and very few households utilised available healthcare services during illness. CHE was also more concentrated among three-fourth of the poorest than the richest households. It was further found that socioeconomic characteristics of the households contributed to CHE. Besides, annually, approximately 2% of households fell into poverty due to CHE; this corresponds to the economic impoverishment of nearly 22,000 people in the *Gedeo* zone, Ethiopia. Such impoverishment due to OOP healthcare payments, in turn, has a major impact on household health and affects the utilisation of healthcare services. Consequently, because of the high risk of financial catastrophe and impoverishment, in turn, achieving universal healthcare coverage could be impossible.

The need for utilisation healthcare services might be higher than the actual utilisation of healthcare services. However, in this study, there were a low number of households with OOP healthcare payments and there were also a low number of households utilising available healthcare services. Those households in the richest quintile sought more healthcare services and had more OOP healthcare payments than those households in the poorest quintile. This was evidenced by OOP healthcare payments resulting in an additional 2% of households falling into poverty. This finding was consistent with other studies from Zimbabwe [43], as poverty was associated with low utilisation of healthcare services. The poorest households suffered from high OOP healthcare payments, which resulted in a higher incidence of CHE. Although the richest households tended to have higher OOP healthcare payments, the capacity to pay for the richest households was also higher than that of the poorest households. In fact, given that some of the poorest households may not seek healthcare due to high OOP healthcare payments, the financial burden could be even higher for the poorest if this factor was accounted for. Therefore, developing a viable removal or reduction of financial risk for the poorest households is critical.

Based on the findings of this study, the lack of financial health protection in the study area may indicate that the financial burden is heavier among the poorest households and has implications for the consumption of essential basic necessities. This might be one of the reasons why poor households allocated a greater share of their household budget to food compared to rich households [44].

Heavy reliance on OOP healthcare payments posed a financial burden on households and lead to different types of coping strategies to be adopted to cover healthcare payments for maternal and neonatal illness. In our study, coping strategies adopted to meet OOP healthcare payments are consistent with a study from rural Bangladesh [45].

Our findings of CHE based on different thresholds were comparable with other studies reported from Rwanda, [46], Kenya [47], Ghana [48], and Uganda [49]. Therefore, OOP healthcare payment impoverishes households and limits the choice of seeking healthcare services during illness.

However, results from this study were not consistent with a previous study in Ethiopia [50], which reported that 24% of households faced financial catastrophe due to OOP healthcare payments, and such catastrophe pushed 5.8% of households into poverty. This difference could be due to that the study participants were with chronic illness, while we were following acute illness among mothers and neonates. Our findings in this regard are similar, implying that 6% of total households faced financial catastrophe and this was three times higher among households whose food expenditure was below the poverty line [51].

The findings of this study also indicated that those poorer households experienced CHE more often. Some studies from Ethiopia [50], Mongolia [51], and Swaziland [44] reported that *households* who were *poor* became even *poorer* after CHE. However, estimation techniques of OOP healthcare payments differed in many studies. For instance, the household’s socioeconomic status was determined using the household’s total expenditure or asset index. Choosing asset quintiles to determine the wealth quintiles in this study was supported by a study from Asia [52].

This study was a population-based study as population-based design and population-based household data are useful to provide empirical literature in assessing patterns and extent of financial risk, in tackling the poverty impact of OOP healthcare payments, and in reducing the financial burden of incurring direct medical and non-medical expenditures for healthcare [53]. A recent *population*-*based* cohort *study* in the Democratic Republic of Congo [53] demonstrated that a population-based design can yield disaggregated data on the medical and non-medical expenditures, and may also improve understanding of the nature of the economic and social hardships experienced by households at a community-level. Besides, in areas where the provision of public services requires effective policing towards improved health equity and service coverage, and in areas in which multiple wealth-related disparities were common, strong primary evidence from population-based studies is essential to inform technical and political decision-makers.

This study possessed certain limitations. The distribution of OOP healthcare payments was heavily skewed rightwards as there were many small values, a few very large ones, and many zero values. As a consequence of this departure from the normal distribution, the frequency with which a conventional confidence interval for the OOP healthcare payment estimate would not capture the true population parameter and may be greater or might be higher than the probability stated for the confidence interval. Besides, our study did not identify those who forwent utilisation healthcare services since they could not afford healthcare payments and therefore did not incur OOP healthcare payments. Moreover, household annual total household expenditure and OOP healthcare payments were self-reported; and thus there may be over or under-reporting. The findings of this study could also be exaggerated. This was because the OOP healthcare payments used in the analysis were the sum of the three categories (i.e., pregnant women, postpartum women, and neonates), the summation of all repeated visits, and we reported it on an annual basis. Furthermore, all expenditures and all OOP healthcare payments from households may not be covered within the questionnaire. This study also focused only on OOP healthcare payments, and it could not measure the impact of opportunity payments, such as income losses during illness, socioeconomic shocks or death. Therefore, the findings from this study should be interpreted with care. A loss to follow-up of study participants include those who were not easily contacted or those who presented with illness after the defined time frame for postpartum and neonatal periods, and may have introduced a selection bias.

However, this study also possessed some key strengths. This study was one of the very few population-based cohort studies to investigate illness incidence or period prevalence with actual OOP healthcare payments. This study also seems to be the first of its kind of analysis of OOP healthcare payments in Ethiopia using a dataset that brings a comprehensive understanding of the three categories. Given that our study was conducted in rural areas, in analysis, household expenditure or consumption data were used instead of income data. This was because formal employment was less common, many households had multiple and/or continually changing sources of income, and home production was more widespread. To keep the validity of the data collection on income and expenditure, a standard questionnaire was used which was commonly employed at the national level and beyond, and the data collection process was a direct measure at the household level. Recall periods also differed for different types of goods. For the goods that were purchased infrequently, we used a sufficiently long period, so that the consumption during the period was representative of the reference period (i.e., in a year). We also used a sufficiently short period for the goods that were purchased and consumed frequently, so that households may remember expenditures and consumption with reasonable accuracy. As a cross-check, household income was also compared with household expenditure aggregates. To maintain the balance of expenditure data, aggregating different components of expenditures was done, and a common reference period was established for all items, e.g., a year.

## Conclusions

This study demonstrated that health inequity in the household’s budget share of total OOP healthcare payments in southern Ethiopia was high. Besides, utilisation of maternal and neonatal healthcare services very low and seeking such healthcare poses a substantial financial risk during illness among rural households. Both catastrophe and impoverishment due to OOP healthcare payments were high, and their proportion to total household expenditure would alert policymakers to take commensurate action. All differences in OOP healthcare payments highlighted the heavier burden borne by the poorest households.

Therefore, the issue of health inequity should be considered when setting priorities to address the lack of fairness in maternal and neonatal health. Health inequities should be reduced by using mixed government policy action on the social determinants of health: including improvement of schooling, an increment of employment, and improvement of socioeconomic status of the households at least in the study area. The health policy implications of this study include: the Ministry of health should intensify the actions to tackle the root causes of ill-health and health inequities, and should continue increasing advocacy to provide essential healthcare services to women and neonates.

To achieve the goal of universal healthcare coverage, mothers and neonates have to get access to prepayment and financial risk pooling mechanisms. These interventions should target the poorest households, particularly in rural areas, in the following ways: reduce reliance on OOP healthcare payments using payments made in advance of illness to treat sick mothers and neonates, and introduce prepayment schemes to cover payments for transportation and subsistence, such as transportation vouchers and conditional cash transfer during an illness of pregnancy, postpartum, and neonatal periods. Financial risk should be pooled in some way and used to fund healthcare services for every mother and neonate who is covered, find alternative sources of financing to exempt or remove all user fees; and make health insurance available.

## Supplementary information

**Additional file 1.**

**Additional file 2.**

**Additional file 3.**

## Data Availability

The data for this paper are available at https://osf.io/prnj9/.

## Abbreviations

ADePT: Automated development economics and poverty tables
CBHI: Community-based health insurance
CHE: Catastrophic health expenditure
CI: Confidence interval
ETB: Ethiopian Birr
FRP: Financial risk protection
LMICs: Low and middle-income countries
MPO: Mean positive overshoot
OOP: Out-of-pocket
PPP: Purchasing power parity
SHI: Social health insurance
SPSS: Statistical package for social sciences
$: United States dollars
