# Supplementary material for: Financial risk of seeking maternal and neonatal healthcare in southern Ethiopia: a cohort study of rural households
Source: Int J Equity Health. 2020 May 18;19:69. doi: 10.1186/s12939-020-01183-7 (PMC7236117; doi:10.1186/s12939-020-01183-7)
Supplement: Supplementary file 1 — Additional file 1. [file 12939_2020_1183_MOESM1_ESM.doc]

Form: 9 Duuchcha fayyuntenna fayyunte labbaxxa yaanuwwa kaddebaaxxae minike hadi’a fulaaka maallaqa borreessatika qortama.

| **Minike hadika ifisanjo** | |
| --- | --- |
| Miniik Laakkossi | --------/-----/------/--------/--------/ |
| Koodde | 2. Ama |
| Oddooli | 1. Mokkoniissa 2/ Tumaata cirracha 3/ Hassee Haro |
| Shiixxok/Gooxe/Hiwwaasse | ------------------------------- |
| Qortumiinixxi Barra | ------------------------------- |
| Qrtuma asseekik summina beessis | |
| Towateekik/Suppervayizeeretik summina beessis | |
| Dhugaa/Maaraja/bukkasse’e aassekik beessinna summi | |
| Qortumiinxxi Gumma   1. Muuxeme’en 2) Siiliinxxi ama mine hexxebaan 3) Duuchching mini hadi he’nebaan /Hexxebaan   4) Welebarra’a adhdhineennen 5) Ginbbe’neen | |

Kuta1: Fayyunte labbabaaxxe yaanuwwa’a minike hadi’a fuleeka maallaqa (Tini mitte yanna’ni wo’mitaaxxen)

| **Lakkoossa** | **Qortumuwwa** | **Koodde** | **Sa’I** |
| --- | --- | --- | --- |
| **Oddichchi Qcinnikonnechin butti’a hedheeke akeeka’ni mini hadi hitteeke maallaqa falichcha’ne?** | | | |
| 901 | Inddaachi’a | ---------------------Maallaqa (Barrate’ni) |  |
| 902 | Uwwenddaaxxe la’uwwa’a (Ifinjjoke’e wode’ekee, Bilibilake’e) | --------------------- Maallaqa /Agenke’ni/ |  |
| 903 | Barachchotee (Oosetee, ilaanchotee ) | ---------------- Maallaqa /Seemisteerete’ne (4  agetnjo) |  |
| 904 | Miniike Kiri’a | ----------------- Maallaqa (Agenjoken’i) |  |
| 905 | Fayyunte heqqeccatee | ------------------ “ (Woggate’ni) |  |
| 906 | Mini giddike miyuwwi’a | -------------------- “ (Woggate’ni) |  |
| 907 | Oddoonotee | ------------------Maallaqa Woggate’ni |  |
| 908 | Qammachchatee Bishikililetee, Gaarretee, Mootoretee, itta ittan) | -------------------- Maallaqa Woggate’ni |  |
| 909 | Mini giddika miyuwwa hire hiissatee (she’luwwannna welewelecho) | ---------------------- Maallaqa Woggate’ni |  |
| 910 | Liqauuawatee (Kulle) | --------------------- Maallaqa Woggate’ni |  |
| 911 | Welewelenna (Kulle) | --------------------- Maallaqa Woggate’ni |  |
| 912 | Sa’eke sase agenji giddo inddaacho mini hadii’ni yookin welebaa’ni alifitinee? | 1. Waawwo’o 1) Eeti |  |
| 913 | Miniinke anni’a woggate’ni aaga’nek maallaq oddichiqicin me’eke? | --------------------- Maallaqa Woggate’ni |  |
| 914 | Mini hadik wggatini alfia’neke maalaqme’eke? | --------------------- Maallaqa Woggate’ni |  |

Forme 9: Duuchcha fayyuntenna fayyunte kaddebaaxxe yaanuwwa’a fulaaka maalaqa borreessatika qortuma

| **Minike hadika ifisanjo** | |
| --- | --- |
| Miniik Laakkossi | 1. --------/-----/------/--------/--------/ |
| Koodde | 1. Ama |
| Oddooli | 1. Laatto anno lakkote kaddoole ^1kkak^ Belti 2. Laatto anno lakkote kaddoole 2^kkak^ belti   1) Mokkoniisa 2/ Tumaata cirracha 3/ Hassee Haro |
| Shiixxok/Gooxe/Hiwwaasse | ------------------------------- |
| Qortumiinixxi Barra | ------------------------------- |
| Qrtuma asseekik summina beessis | |
| Suppervayizeeetik “ “ | |
| Dhugaa bukkasse aassekik summinna beessis | |
| Qortumiinxxi Gumma | |
| 1. Muuxeme’en 2) Siiliinxxi ama mine hexxebaan 3) Duuchiing minik hadi hedhebaan  4. Welebarra’a adhdhineennen 5) Ginbbe’neen | |
| Dhibo heene yanna’ite 1) Ilatiedon 2) Ilatixxe yanna’niten 3) Ilati uduman 4) Laattoannoxxe  yanna’niten. | |
| Saxxeexxe 2 torbbagiddo atixxee yookin beltiinxxe dhibo’a ci’lo alffatee habatemma me’iteetak?  1. Mootummatixxe orggoba  2. Gilletixxe orggoba | |

Kuta 2: Saxxeexxe 2 Torbbagiddo Illeiima hinggan’I ci’lo alffatee fuleeke maalaqika qortuma

| **Laakoossa** | **Qortumuwwa** | **Koodde** | **Sa’I** |
| --- | --- | --- | --- |
| 915 | Me’ele ifiaa yookin beltokee ci’lp alffatee me’itinee?  Illeiima hige ci’lo alffatee itt aane hedheeke jeej i’a maa chchofulichitine? | --------------------------- |  |
| 916 | Qorissakee | ----------------------------Maallaqa |  |
| 917 | Uudenshshatee | ----------------------------Maallaqa |  |
| 918 | Mari’etee/kaardetee | ----------------------------Maallaqa |  |
| 918 | Me’e higatixxe transpoortetee | ----------------------------Maallaqa |  |
| 920 | Inddaachi’a/ Adoteenna welechi’a | ----------------------------Maallaqa |  |
| 921 | Lebemeexxi kookkisaakee fuleek maallaqi | ----------------------------Maallaqa |  |
| 922 | Aagiyyo hasissasa’na gophphetek maalagi? | ----------------------------Maallaqa |  |
| 923 | Aadatixxe ci’lo’a fuleek maallaqi? | ----------------------------Maallaqa |  |
| 924 | Welewelechi’anna (Kulle) | ----------------------------Maallaqa |  |
| 925 | Duuchcha fuleekmaalaaqi | ----------------------------Maallaqa |  |
| 926 | Ci’lo alffatee hitteexxe yanna birittete? | ------------------------Yanna/sate) |  |
| **Kuta3: Fulekmailla dande’atiki ori** | | | |
| 927 | Liqa liqeeffateete? | 1. Waawwo’o 1. Eeti | Waawwote kaddoole 923 ba’a sa’i |
| 928  929 | Hittee qico liqeeffaeete?  Ayyenaani liqeeffattefe? | ----------------------------Maallaqa   1. Minihadinaa’ni 2. Lla’akee’ni /jaalok’eni 3. Welebaa’ni |  |
| 930 | Wolade Affedhemma’a? | 1. Waawwo’o 1. Eeti | Waawwote kaddoole 932 ba’a sa’i |
| 931 | Hittee qicomma’a liqeeffatetek? | ----------------------------Maallaqa |  |
| 932 | 927 “waawwote kaddoole, cil’o alffatee karra gurgareete? | 1. Waawwo’o 1. Eeti | Waawwote kaddoole 934 ba’a sa’i |
| 933 | Ci’lotixxe daafe’a hitettexxi gurgurtetexxi karra maachchomma’a? | 1. Boga 2. Minika haro 3. Kaamole, Gaara ittaittaa 4. Minika miyawasa 5. Welewelecho |  |

Kuta 4: Saxxeexxe 2 Torbba giddo diiphphe ci’lo alffatee fuleeka maallaqa hubachchotika qortuma

| **Laakkossa** | **Qortumuwwa** | **Koodde** | **Sa’i** |
| --- | --- | --- | --- |
| 934 | Me’ele atinna beltiatika dicnphphineexxi ci’lo alffitine? | --------------------------- |  |
| **Diinphphe ci’lo aliffatee butti’ni kulemeeke jeeja’ni hittee maallalaqa fulichitete?** | | | |
| 935 | Diinphpheexxi ci’lo alffate? | ---------------------Maallaqa |  |
| 936 | Diinphphachi’a Ali ggakee/ Taqetee? | ---------------------Maallaqa |  |
| 937 | Qorssake’e? | ---------------------Maallaqa |  |
| 938 | Uudenshshatee? | ---------------------Maallaqa |  |
| 939 | Inddachi’anna ha’winaachi’a? | ---------------------Maallaqa |  |
| 940 | Genne higatixxe mammarensha’a? | ---------------------Maallaqa |  |
| 941 | Aliffa gohphetek maalaqi? | ---------------------Maallaqa |  |
| 942 | Lebeme fuleeke maallaqi qarqaarakee? | ---------------------Maallaqa |  |
| 943 | Welewelechuwwi’a | ---------------------Maallaqa |  |
| 944 | Duuchchi fuleek maallaqi | ---------------------Maallaqa |  |
| **Kuta 5: Fuleeka maallaqa dande’atike ra** | | | |
| 945 | Liqa liqeeffateta’a? | 0. Waawwo’o 1. Eeti | Waawwote kaddoole 949 ba’a sa’i |
| 946 | Hitteeqico lieeffateete? | ---------------------Maallaqa |  |
| 947 | Ayyenaa’nitee liqeeffatettek? | 1. Minihadii’ni 2. Olla’akeeni /Jaaloke’eni/ 3. Welebaa’ni |  |
| 948 | Wolade afeemma’a liqeeffatinee’k maallaqi? | 0. Waawwo’o 1. Eeti | Waawwote kaddoole 950 ba’a sa’i |
| 949 | Hittee qicomma’a wolade? | ---------------------Maallaqa |  |
| 950 | “945”Waawwote kaddoole, ci’lotee hitineexxi karra qurgutinee? | 0. Waawwo’o 1. Eeti | Waawwote kaddoole 951 ba’a sa’i |
| 951 | Ci’lotixxe dafe’a hitineexxi gurgutinexxi karra maachecho? | 1. Boga  2. Minike hore  3. Kaamole, Gaarre,itta ittann  4. Minigiddika miyuwwan  5. Welechchon |  |

**Galateeffatannon**
