# Supplementary material for: Financial risk of seeking maternal and neonatal healthcare in southern Ethiopia: a cohort study of rural households
Source: Int J Equity Health. 2020 May 18;19:69. doi: 10.1186/s12939-020-01183-7 (PMC7236117; doi:10.1186/s12939-020-01183-7)
Supplement: Supplementary file 2 — Additional file 2. [file 12939_2020_1183_MOESM2_ESM.doc]

**ፎርም 9: ጠቅላላ ጤናና ጤና ነክ ያልሆኑ የቤተሰብ ወጪ መመዝገቢያ መጠይቅ**

| **የቤተሰብ መግለጫዎች** | |
| --- | --- |
| የቤት ቁጥር | [____/____/___/____/____/] |
| ኮድ | 2. እናት |
| ቀበሌ | 1) መኮኒሳ 2) ቱማታ ጨሪቻ 3)ሀሴ ሀሮ |
| ንዑስ ወይም ጎጥ ወይም ህዋስ | ........................................... |
| የቃለ መጠይቅ ቀን | ቀን..................................... |
| ቃለ መጠይቅ አድራጊ ስምና ፊርማ.......................................... | |
| ሱፐርቫይዘር ስምና ፊርማ..................................................... | |
| መረጃ አስገቢ ስምና ፊርማ...................................................... | |
| የቃለ መጠይቁ ዉጤት:  1. ተጠናቀቀ 2. እርጉዝ እና~ ቤት የለችም 3. ሁሉም የቤተሰቡ አባላት ቤት የሉም 4. ሌላ ቀጠሮ ወስደናል 5. እንቢ ብለዋል | |

| **ክፍል 1. ጤና ነክ ያልሆኑ የቤተሰብ የወጣ ወጪ (ይህ ለአንድ ጊዜ የሚሞላ ነዉ)** | | | |
| --- | --- | --- | --- |
| ቁጥር | ጥያቄዎች | ኮድ | ዝለል |
|  | በአማካይ ቤተሰብሽ በሚከተሉት ላይ ምን ያህል ወጪ ያወጣል? | | |
| 901 | ለምግብ የሚከተሉትን ለምሳሌ |  |  |
| A | ቤተሰብዎ ምግብ ለመግዛት ምን ያህል ያወጣል? (ሰብሎች, ጤፍ, ዘይት, ጨው, ወዘተ ...) | .................ብር/በቀን |  |
|  | ቤተሰብዎ ከእርሻ ወይም በቤት ዉስጥ የሚመረቱ ምግብ ይጠቀማል? | 1. አይ 1. አዎ | አይ ከሆነ ወደ b ዝልል |
|  | አዎ ከሆነ, የተመረተው የምግብ ቢሸጥ ምን ያህል ያወጣል? | .................ብር/በቀን |  |
| B | ቤተሰብዎ ለሰራዉ ስራ እንደክፍያ የሚያገኘዉ የምግብ እህል አለ? | 1. አይ 1. አዎ | አይ ከሆነ ወደ c ዝልል |
|  | አዎ ከሆነ, የሚሰጠዉ የምግብ እህል ቢሸጥ ምን ያህል ያወጣል? | .................ብር/በቀን |  |
| C | ቤተሰብዎ እንደ ስጦታ ወይም ብድር የሚቀበለው የምግብ እህል አለ? | 1. አይ 1. አዎ | አይ ከሆነ ወደ d ዝልል |
|  | አዎ ከሆነ, እንደ ስጦታ ወይም ብድር የሚየገኙት የምግብ መጠን ቢሸጥ ምን ያህል ያወጣል? | .................ብር/በቀን |  |
| D | ቤተሰብዎ ምግብ ወይም የምግብ እህል ለሌላ ቤተሰብ ይሰጣል? | 1. አይ 1. አዎ | አይ ከሆነ ወደ e ዝልል |
|  | አዎ ከሆነ, የምግብ መጠን ቢሸጥ ምን ያህል ያወጣል? | .................ብር/በቀን |  |
| E | ቤተሰብዎ ምግብ ለማብሰልና ለማገዶ ምን ያህል ገንዘብ ያወጣል? | .................ብር/በቀን |  |
|  | ጠቅላላ ወጪ ለምግብ | .................ብር/በቀን |  |
| 902 | ለአገልግሎት ክፍያዎች (ለመብራት፣ ለዉሃ፣ለስልክ) | .................ብር በወር |  |
| 903 | ለትምህርት (ለልጆች፣ ለእራስ) | ..........ብር በሴሚስተር (4ወር) |  |
| 904 | ለቤት ኪራይ | .................ብር በወር |  |
| 905 | ጤና ለመጠበቅ | .................ብር ባለፈዉ 3 ወር |  |
| 906 | ለቤት ቁሳቁስ መገልገያዎች | .................ብር በአመት |  |
| 907 | ለልብስ | .................ብር በአመት |  |
| 908 | ለጥገና (ብስክሌት፣ጋሪ፣ሞተር ወዘተ) | .................ብር በወር |  |
| 909 | የቤት ዉስጥ መገልገያዎች ለመተካት (ምጣድ ወዘተ) | .................ብር በወር |  |
| 910 | ብድር ለመክፍል (ጥቀሱ) | .................ብር በወር |  |
| 911 | ሌሎች (ጥቀሱ) | .................ብር በወር |  |
| 912 | ባለፈዉ 3 ወር ዉስጥ፣የምግብ ስጦታ ከቤተሰብ ወይም ከሌላ ሰዉ አግኝታችል? | 1. አይ 1. አዎ |  |
| 913 | የቤተሰቡ አባወራ አመታዊ ገቢዉ በአማከይ ስንት ነዉ? | .................ብር |  |
| 914 | የቤተሰቡ አባላት የሁሉም አመታዊ ገቢዉ በአማከይ ስንት ነዉ? | .................ብር |  |

አመሰግናለሁ!!!

**ፎርም 9: ጠቅላላ ጤናና ጤና ነክ ያልሆኑ የቤተሰብ ወጪ መመዝገቢያ መጠይቅ**

| **የበተሰብ መግለጫዎች** | |
| --- | --- |
| የቤት ቁጥር | [____/____/___/____/____/] |
| ኮድ | 2. እናት  3. ጨቅላ ህፃን መንትያ ከሆነ 1ኛ ልጅ  4. ጨቅላ ህፃን መንትያ ከሆነ 2ኛ ልጅ  4. ጨቅላ ህፃን መንትያ ከሆነ 3ኛ ልጅ |
| ቀበሌ | 1) መኮኒሳ 2) ቱማታ ጨሪቻ 3)ሀሴ ሀሮ |
| ንዑስ ወይም ጎጥ ወይም ህዋስ | ........................................... |
| የቃለ መጠይቅ ቀን | ቀን..................................... |
| ቃለ መጠይቅ አድራጊ ስምና ፊርማ.......................................... | |
| ሱፐርቫይዘር ስምና ፊርማ..................................................... | |
| መረጃ አስገቢ ስምና ፊርማ...................................................... | |
| የቃለ መጠይቁ ዉጤት:  1. ተጠናቀቀ 2. እርጉዝ እና~ ቤት የለችም 3. ሁሉም የቤተሰቡ አባላት ቤት የሉም 4. ሌላ ቀጠሮ ወስደናል 5. እንቢ ብለዋል | |
| ህመሙ ምንጊዜ ነዉ? 1. ቅድመ ወሊድ 2. በወሊድ ወቅት 3. ድህረ ወሊድ 4. ጨቅላ ህፃን | |

ባለፈዉ 2 ሳምንት ዉስጥ፣ ለራስሽ ወይም ለልጅሽ ለመታከም የት ነበር የሄድሽዉ?

1. የመንግስት
2. የግል

| **ክፍል 2. ባለፈዉ 2 ሳምንት ዉስጥ፣ ለተመላላሽ ህክምና የወጣ ወጪ መጠይቅ** | | | |
| --- | --- | --- | --- |
| ቁጥር | ጥያቄዎች | ኮድ | ዝለል |
|  | ስንት ጊዜ ለራስሽ ወይም ለልጅሽ ለመታከም ሄደሻል? | ................. |  |
|  | ምን ያህል ወጪ ለሚከተሉት ተመላላሽ ህክምና አወጣሽ? | | |
|  | ለመድሃኒት? | ..................(ብር) |  |
|  | ለምርመራ? | ..................(ብር) |  |
|  | ለማማከር? ለካርድ | ..................(ብር) |  |
|  | ለትራንስፖርት? (ደርሶ መልስ) | ..................(ብር) |  |
|  | ለምግብ? (ተጨማሪ ምግቦች፣ፈሳሽ፣ወተት፣ ወዘተ) | ..................(ብር) |  |
|  | ተጨማሪ ወጪ ለተንከባከቢ? | ..................(ብር) |  |
|  | ያጣሽዉ ገቢ? | ..................(ብር) |  |
|  | ለባህላዊ ህክምና? | ..................(ብር) |  |
|  | ሌሎች (ጥቀሱ) | ..................(ብር) |  |
|  | ጠቅላላ ወጪ | ..................(ብር) |  |
|  | ህክምና ለማግኘት ምን ያህል ጊዜ አጠፋሽ? | ..................(ሰአት) |  |
| **ክፍል 3. ወጪን የመggሚያ መንገድ** | | | |
|  | ብድር ተበድረሻል? | 0. አይደለም 1.አዎ | አይደለም ከሆነ፣ ወደ 932ዝለል |
|  | ምን ያህል? | ..............ብር |  |
|  | ከማን ተበደርሽ? | 1. ከቤተሰብ 2. ከጎረቤት/Òደኛ 3. ሌላ...................... |  |
|  | ወለድ ነበረዉ? | 0. አይደለም 1.አዎ | አይደለም ከሆነ፣ ወደ 932 ዝለል |
|  | ምን ያህል? | ..............ብር |  |
|  | 927 አይደለም ከሆነ፣ ለመታከም ንብረት ሸጠሻል? | 0. አይደለም 1.አዎ | አይደለም ከሆነ፣ ወደ 934 ዝለል |
|  | የንብረቱ አይነት? | 1. መሬት 2. የቤት እንሰሳ 3. መኪና, ጋሪ, ወዘተ 4. የቤት ቁሳቁስ 5. ሌላ...................... |  |

አመሰግናለሁ!!!

| **ክፍል 4. ባለፈዉ 2 ሳምንት ዉስጥ፣ ለተኝቶ ህክምና የወጣ ወጪ መጠይቅ** | | | |
| --- | --- | --- | --- |
| ቁጥር | ጥያቄዎች | ኮድ | ዝለል |
|  | ስንት ጊዜ ለራስሽ ወይም ለልጅሽ ተኝተሸ ታከመ/ታከምሽ? | ................. |  |
|  | ለተኝቶ ህክምናዉ ለሚከተሉት ምን ያህል ወጪ አወጣሽ? | | |
|  | ተኝቶ ለመታከም? | ..................(ብር) |  |
|  | ለአልጋ? | ..................(ብር) |  |
|  | ለመድሃኒት? | ..................(ብር) |  |
|  | ለምርመራ? | ..................(ብር) |  |
|  | ለምግብ? (ተጨማሪ ምግቦች፣ፈሳሽ፣ወተት፣ ወዘተ) | ..................(ብር) |  |
|  | ለትራንስፖርት? (ደርሶ መልስ) | ..................(ብር) |  |
|  | ያጣሽዉ ገቢ? | ..................(ብር) |  |
|  | ተጨማሪ ወጪ ለተንከባከቢ? | ..................(ብር) |  |
|  | ሌሎች (ጥቀሱ) | ..................(ብር) |  |
|  | ጠቅላላ ወጪ | ..................(ብር) |  |
|  | ተኝቶ ህክምና ለማግኘት ለሚከተሉት ምን ያህል ጊዜ አጠፋሽ? | | |
|  | በጉዞ ላይ | ..................(ሰአት) |  |
|  | ተኝቶ ህክምናዉ ስንት ቀን ነበር? | ..................(ቀን) |  |
| **ክፍል 5. ወጪን የመggሚያ መንገድ** | | | |
|  | ብድር ተበድረሻል? | 0. አይደለም 1.አዎ | አይደለም ከሆነ፣ ወደ 952 ዝለል |
|  | ምን ያህል? | ..............ብር |  |
|  | ከማን ተበደርሽ? | 1. ከቤተሰብ 2. ከጎረቤት/Òደኛ 3. ሌላ...................... |  |
|  | ወለድ ነበረዉ? | 0. አይደለም 1.አዎ | አይደለም ከሆነ፣ ወደ 952 ዝለል |
|  | ምን ያህል? | ..............ብር |  |
|  | 947 አይደለም ከሆነ፣ ለመታከም ንብረት ሸጠሻል? | 0. አይደለም 1.አዎ | አይደለም ከሆነ፣ 953ን ዝለል |
|  | የንብረቱ አይነት? | 1. መሬት 2. የቤት እንሰሳ 3. መኪና, ጋሪ, ወዘተ 4. የቤት ቁሳቁስ 5. ሌላ...................... |  |

አመሰግናለሁ!!!
