# Supplementary material for: Financial risk of seeking maternal and neonatal healthcare in southern Ethiopia: a cohort study of rural households
Source: Int J Equity Health. 2020 May 18;19:69. doi: 10.1186/s12939-020-01183-7 (PMC7236117; doi:10.1186/s12939-020-01183-7)
Supplement: Supplementary file 3 — Additional file 3. [file 12939_2020_1183_MOESM3_ESM.doc]

**Form 9: Total health and non-health expenditures of households**

| **Household characteristics** | |
| --- | --- |
| House ID | [____/____/___/____/____/] |
| ID | 2. Woman |
| Kebele/village (Name) | 1) Mekonisa 2) Tumata chiricha 3)Hase haro |
| Sub-kebele |  |
| Date interview | Date..................................... |
| Interviewer name and signature.......................................... Supervisor name and signature........................................... | |
| Data clerk name and signature............................................ | |
| Result of interview: 1. Completed 2. No mother at home 3. No all family at home 4. Another appointment 5. Refused | |

| **Section 1. Household expenditures on non-health consumptions [This section is only necessary to ask once]** | | | |
| --- | --- | --- | --- |
| **No.** | **Questions and Filters** | **Coding Categories** | **SKIP** |
|  | On average how much did your household spend on the following items? | | |
|  | Food and supplies (e.g. plates, cups etc.) |  |  |
| a | How much did your household spend to buy food? (Cereals, teff, oil, salt, etc...) | ……….birr/ day |  |
|  | Did you consume food that was **grown or produced** by the household? | 1. No 1. yes | If no skip to b |
|  | If yes, how much would it cost to buy the quantity of food that was grown or produced? | ……….birr/day |  |
| b | Did you consume food that was **received as wages** in kind for work? | 1. No 1. yes | If no skip to c |
|  | If yes, how much would it cost to buy the quantity of food that was received as wages in kind for work? | ……….birr/day |  |
| c | Did you consume food that was **received as a gift or loan?** | 1. No 1. yes | If no skip to d |
|  | If yes, how much would it cost to buy the quantity of food that was received as gift or loan? | ……….birr/day |  |
| d | Did you give away food outside of the household? | 1. No 1. yes | If no skip to e |
|  | If yes, how much would it cost to buy the quantity of food that was given away? | ……….birr/day |  |
| e | About how much money does your household spend on **cooking and lighting Fuel**? | ……….birr/day |  |
|  | **Total expenditure on food and supplies** | **……….birr/ day** |  |
|  | Utilities (electricity, water , telephone) | ……….birr /month |  |
|  | Education (School for children or self) | ……….birr/ term (4 months) |  |
|  | House rent | ……….birr/ month |  |
|  | Health care (for the household) | ……….birr in last three months |  |
|  | Goods and utensils for the household use | ……….birr /year |  |
|  | Clothes | ……….birr /year |  |
|  | Maintenance of bicycle, cart, motor bike | ……….birr/ month |  |
|  | Replacements of household appliances (stove, lanterns, etc.) | ……….birr/ month |  |
|  | Reimbursement of loan (describe) | ……….birr/ month |  |
|  | Others (describe) | ……….birr / month |  |
|  | Have you received any in kind food item as a gift from relatives or others during the last 3 months? | 1. No 1. Yes |  |
|  | What is the total annual income for the head of your household (in average)? | ....................Birr |  |
|  | What is the total annual income for all the members of your household (in average)? | ....................Birr |  |

Thank you!!!

**Form 9: Total health and non-health expenditures of households**

| **Household characteristics** | |
| --- | --- |
| House ID | [____/____/___/____/____/] |
| ID | 2. Woman  3. Single  4. If twins, to second child  5. If triplet, to third child |
| Kebele/village (Name) | 1) Mekonisa 2) Tumata chiricha 3)Hase haro |
| Sub-kebele |  |
| Date interview | Date..................................... |
| Interviewer name and signature.......................................... Supervisor name and signature........................................... | |
| Data clerk name and signature............................................ | |
| Result of interview: 1. Completed 2. No mother at home 3. No all family at home 4. Another appointment 5. Refused | |
| Ill during? 1) ANC follow-up 2) delivery 3) postnatal period 4) Neonatal period | |

In the last 2 weeks, for you/your child illness, where did you get the treatment?

1. Government health facility
2. Private health facility

| **Section 2. In the last 2 weeks, Out-patient care expenditures** | | | |
| --- | --- | --- | --- |
| **No.** | **Questions and Filters** | **Coding Categories** | **SKIP** |
|  | How many times //have you//has your child// visited a health facility for out-patient care due to any illness since your child was born? | **...............** |  |
|  | For the last out-patient visit(clinics or OPD in hospitals):  How much did you spend on out-patient care for treatment of the illness for//you//your// child? | | |
|  | Drug | .....................Birr |  |
|  | Investigation/tests | .....................Birr |  |
|  | Consultation fee/card | .....................Birr |  |
|  | Transport to and From health facility | .....................Birr |  |
|  | Extra food cost (any special food bought for the infant for example any milk or other food, fluid and food bought for caregivers who accompanied the infant to the treatment provider) | .....................Birr |  |
|  | Additional expenses for care giver | .....................Birr |  |
|  | Wages lost | .....................Birr |  |
|  | Traditional healer | .....................Birr |  |
|  | Others (describe) | .....................Birr |  |
|  | Total expenditure | .....................Birr |  |
|  | For the last out-patient visit (clinics or OPD in hospitals):  How much time did you spend on out-patient care for treatment of the illness for //you//your// child? (This includes time travelling back and forth and time spent at the facility) | .....................hours |  |
| **Section 3. Expenditure coping mechanism** | | | |
|  | Did you loan to cover your expense? | 0. No 1. Yes | If No, SKIP to 932 |
|  | How much? (Birr) | ........................ |  |
|  | From whom did you loan? | 1. Family 2. Neighbour/friend 3. Other (mention) |  |
|  | Does it have any interest? | 0- No 1.Yes | If No, SKIP to 932 |
|  | How much? (Birr) |  |  |
|  | If no in 927, 930, did you sell your property to get treatment? | 0. No 1. Yes | If No, SKIP to 934 |
|  | What type? | 1. Land 2. Domestic animals 3. Vehicle, cart, etc 4. Household property 5. Other (mention) |  |

Thank you!!!

| **Section 4. In the last 2 weeks, In-patient care expenditures** | | | | |
| --- | --- | --- | --- | --- |
| **No.** | **Questions and Filters** | **Coding Categories** | **SKIP** | |
|  | How many times //have you//has your child// been hospitalised? | **...............** |  | |
|  | For the last hospitalization, how much did you spend on each of the following items during the total stay? | | | |
|  | Admission fee | .....................Birr | |  |
|  | Hospital bed | .....................Birr | |  |
|  | Drug | .....................Birr |  | |
|  | Investigation/tests | .....................Birr |  | |
|  | Food (any special food bought for the infant for example any milk or other food, fluid and food bought for caregivers who accompanied the infant to the treatment provider) |  |  | |
|  | Transport to and From health facility | .....................Birr |  | |
|  | Wages lost | .....................Birr |  | |
|  | Additional expenses for care giver | .....................Birr |  | |
|  | Others (describe) | .....................Birr |  | |
|  | Total expenditure | .....................Birr |  | |
|  | For each of the in-patient visits (clinics or OPD in hospitals):  How much time did you spend on in-patient care for treatment of the illness for //you//your// child? | | | |
|  | Hours spent travelling: | .....................hours |  | |
|  | Days spent in hospital: | .....................days |  | |
| **Section 5. Expenditure coping mechanism** | | | | |
|  | Did you loan to cover your expense? | 0. No 1. Yes | If No, SKIP to 952 | |
|  | How much? (Birr) |  |  | |
|  | From whom did you loan? | 1. Family 2. Neighbour/friend 3. Other (mention) |  | |
|  | Does it have any interest? | 0- No 1.Yes | If No, SKIP to 952 | |
|  | How much? (Birr) |  |  | |
|  | If no in 947, 952, did you sell your property to get treatment? | 0. No 1. Yes | If No, SKIP 953 | |
|  | What type? | 1. Land 2. Domestic animals 3. Vehicle, cart, etc 4. Household property 5. Other (mention) |  | |

Thank you!!!
